# *Candida albicans* Adaptation on Simulated Human Body Fluids under Different pH

**DOI:** 10.3390/microorganisms8040511

**Published:** 2020-04-03

**Authors:** Ana Barbosa, Daniela Araújo, Eduarda Ribeiro, Mariana Henriques, Sónia Silva

**Affiliations:** LIBRO—Laboratório de Investigação em Biofilmes Rosário Oliveira, CEB—Centre of Biological Engineering, University of Minho, 4710-057 Braga, Portugal; pg38833@alunos.uminho.pt (A.B.); danieira.araujo@gmail.com (D.A.); eduardaprr@gmail.com (E.R.); mcrh@deb.uminho.pt (M.H.)

**Keywords:** candidiasis, pathogenicity, environmental cues, virulence factor

## Abstract

*Candida albicans* remains the most problematic of all *Candida* species, causing severe infections. Adaptation to different human body niches, such oral and urinary tracts, has been shown to be essential for survival and critical for virulence of *C. albicans.* Thus, the present work aimed to study the behaviour of *C. albicans* on simulated human body fluids (artificial saliva and urine) at different values of pH (pH 5.8 and 7) by determining its ability to develop two of the most important virulence factors: biofilms and filamentous forms. Under this study, it was demonstrated that *C. albicans* was able to grow as free cells and to develop biofilm communities composed of multiple cell types (yeast and elongated hyphal cells) on both simulated human body fluids and under different pH. It was interesting to note that the pH had little impact on *C. albicans* planktonic and biofilm growth, despite influencing the development of filamentous shapes in artificial saliva and urine. So, it was possible to infer that *C. albicans* presents a high plasticity and adaptability to different human body fluids, namely saliva and urine. These can be the justification for the high number of oral and urinary candidiasis in the whole world.

## 1. Introduction

Yeast species of the genus *Candida* are responsible for 70–90% of human fungal infections, with *Candida albicans* accounting for approximately 40% of all yeast isolates from clinical samples [1,2,3,4]. Infections with *C. albicans* represent an important public health challenge with high economic and medical relevance due to the increased costs of care, time of hospitalisation and high levels of morbidity and mortality, especially in immunocompromised patients [5].

Indeed, *C. albicans* has been described as able to colonize anatomically distinct sites including the urinary [6] and oral tract [7,8]. The pathogenicity of *C. albicans* has been attributed to several virulence factors, such as the ability to evade host defences, adherence, biofilm formation and development of filamentous forms [9,10,11].

The cell responses to environmental changes, such as pH, allow *C. albicans* to take advantage of impaired immunity in debilitated patients and therefore facilitate the establishment of candidiasis [12,13]. The diverse niches that *C. albicans* inhabit vary prominently with respect to environmental pH. For instance, the pH of the oral environment varies significantly resulting from changes in diet, the metabolism of other constituents of the microbiota and salivary flow [14]. In the urinary tract, changes in the amount or type of acid produced are patient-dependent with a urine pH ranging from 4.5 to 8 [15].

Adaptation to different human body niches, such the oral and urinary tracts, has been shown to be essential for survival and critical for virulence in many commensals’ pathogens, namely in *C. albicans* [16,17]. Thus, the present work aimed to study the behaviour of *C. albicans* on simulated human body fluids (artificial saliva and urine) at different pH (5.8 and 7) determining its ability to develop virulence factors, namely, to form biofilms and to develop filamentous forms.

## 2. Materials and Methods

### 2.1. Simulated Body Fluids

To mimic the human body fluids, artificial urine (AU) and saliva (AS), were using during this work. AU (pH 5.8 and 7) and AS (pH 5.8 and 7) were prepared with slight modifications to that described by Silva et al. (2010) and (2013), respectively [18,19]. The composition of AU was CaCl_2_ (0.65 g/L), MgCl_2_ (0.65 g/L), NaCl (4.6 g/L), Na_2_SO_4_ (2.3 g/L), Na_3_C_3_H_5_O(CO_2_)_3_ (0.65 g/L), Na_2_C_2_O_4_ (0.02 g/L), KH_2_PO_4_ (2.8 g/L), KCl (1.6 g/L), NH_4_Cl (1.0 g/L), urea (25 g/L), creatinine (1.1 g/L), and glucose (3 g/L); and the composition of AS was yeast extract (2 g/L), peptone (5 g/L), glucose (2 g/L), mucin (1 g/L), NaCl (0.35 g/L), CaCl_2_ (0.2 g/L), and KCl (0.2 g/L). The pH of simulated body fluids was adjusted with a pH meter (C1010 Benchtop pH Meter, Cleaver Scientific) using hydrochloric acid and/or sodium hydroxide. Roswell Park Memorial Institute (RPMI; Sigma, St Louis, USA) buffered with 3-(N-Morpholino) propanesulfonic acid (MOPS; Sigma-Aldrich, St Louis, MO, USA) and adjusted to pH 7 with sodium hydroxide was used as control of *C. albicans* growth and filamentation in all experiments.

### 2.2. Organism and Growth Conditions

*Candida albicans* SC5314 strain was used in this study. This strain belongs to the Biofilm group collection, located at the Centre of Biological Engineering of Minho University (Braga, Portugal), and its identity was confirmed by PCR-based sequencing with specific primers (ITS1 and ITS4) [20]. For all experiments, cells were subcultured on sabouraud dextrose agar (SDA; Merck, Germany) and incubated for 24 h at 37 °C. An inoculum, obtained from SDA plates, was resuspended in sabouraud dextrose broth (SDB; Merck, Germany) and incubated at 37 °C for 18 h at 120 rpm. After this time, the cells’ suspension was centrifuged for 10 min at 3000 g and 4 °C and washed twice with phosphate-buffered saline (PBS; pH 7, 0.1 M). Pellets were resuspended in 5 mL of PBS and the cellular density adjusted for each experiment using a *Neubauer* chamber (Marienfild, Land-Konicshofem, Germany) to 1 × 10^6^ cells ml^−1^.

### 2.3. Planktonic Growth Analysis

#### 2.3.1. Growth Curves Determination

For the planktonic growth, a cellular suspension of *C. albicans* SC5314 prepared in RPMI, AS (pH 5.8 and 7) and AU (pH 5.8 and 7) were placed in 25 mL Erlenmeyer flasks and incubated for 30 h at 37 °C under agitation in an orbital shaker at 120 rpm. The optical density (OD) at 690 nm was measured over the time using a microtiter plate reader (Thermo Scientific^TM^ Multiskan^TM^ FC, Thermo Fisher Scientific, Finland). The specific growth rate was determined for each condition through the linear regression determination obtained from each specific growth performance. All experiments were performed in triplicate and in a minimum of three independent assays.

#### 2.3.2. Colony Forming Unit (CFU) Quantification

The number of cultivable cells after 24 h of planktonic growth was determined by CFUs enumeration [11]. Thus, from each condition 1 mL of suspension was recovered and the pellets were washed in PBS. Serial dilutions (in PBS) were performed, plated onto SDA and then incubated at 37 °C. The total CFUs per mL (log_10_ CFUs/mL) were enumerated after 24 h of incubation. All experiments were performed in triplicate and in a minimum of three independent assays.

#### 2.3.3. Metabolic Activity Determination

An tetrazolium (XTT) reduction assay was used to determine *C. albicans* cells metabolic activity [11]. For that, after 24 h of grown, 1 mL of each suspension was harvested by centrifugation at 3000 *g* at 4 °C and washed once with PBS. An aliquot of 200 µL of a mixture of 100 µg/mL XTT (2,3-(2-methoxy-4-nitro-5-sulphophenyl)-5-[(phenylamino) carbonyl]-2H-tetrazolium hydroxide) (Sigma-Aldrich, USA) and 10 µg/mL PMS (Sigma-Aldrich, USA) was added to each cells pellet and incubated at 37 °C for 3 h in the dark at 120 rpm. Following this, the OD was measured at 490 nm using a microtiter plate reader. The metabolic activity was compared for each fluid and the absorbance values were standardized per number of CFUs determined previously, as described in Section 2.3.2 (Abs 490 nm/log_10_ CFUs). All experiments were performed in triplicate and in a minimum of three independent assays.

#### 2.3.4. Filaments Enumeration

*Candida albicans* cells were also evaluated in terms of filamentous forms development. For that, at 6, 10 and 24 h, aliquots of each cellular suspension were diluted in PBS and the filaments were counted in an optical microscope using a *Neubauer* chamber. The results were presented as percentage of filamentous forms. In parallel, the morphology of cells was confirmed through fluorescence microscope (Olympus BX51 coupled with a DP71 digital camera; Olympus, Tokyo, Japan), after staining the cells with 1% (*v/v*) of calcofluor (Sigma-Aldrich, EUA) for 15 min in the dark. The excitation line 405 and the emission filters BA 430–470 (blue channel) were used, and images were acquired with the program FluoView FV100 (Olympus). The length of the filaments was also determined using the ImageJ Plug-in (Maryland, USA) software. All experiments were performed in triplicate and in a minimum of three independent assays.

### 2.4. Biofilm Growth Analysis

#### 2.4.1. Biofilm Formation

In order to develop *C. albicans* biofilms, 1 mL of cellular suspension (1 × 10^6^ cells), prepared in RPMI, AS and AU was added to 24-well polystyrene microtiter plates (Orange Scientific, Braine- l’Alleud, Belgium) and incubated at 37 °C and 120 rpm. After 6, 10 and 24 h of incubation, the culture medium was removed and the biofilms were washed twice with PBS to remove the non-adherent cells, and subsequently characterized.

#### 2.4.2. Biofilm Characterization

##### CFUs Quantification

The number of cultivable cells on biofilms was determined by CFUs counting methodology [11]. Briefly, washed biofilms were scraped from the microtiter wells with 1 mL of PBS and the suspensions were sonicated for 10 s at 30% (Ultrasonic Processor; Cole-Parmer, Illinois, USA) to disaggregate the cells from matrix [11]. Serial decimal dilutions of recovered cells in PBS were plated on SDA and incubated for 24 h at 37 °C. The results were presented as total of CFUs and the values were standardized per unit area of well (log_10_ CFUs cm^−2^). All experiments were performed in triplicate and in a minimum of three independent assays.

##### Metabolic Activity Determination

After 24 h of pre-formed biofilms, they were washed and 200 µL of a mixture of XTT and PMS was added at each well and incubated at 37 °C for 3 h in dark at 120 rpm. It was also added to the control wells (to measure background XTT levels). The evaluation was performed as described previously in Section 2.3.3. The metabolic activity was compared for each fluid and the absorbance values were standardized per values of CFUs (Abs 490 nm/log_10_ CFUs). The results were presented as previously described in Section 2.3.3. All experiments were performed in triplicate and in a minimum of three independent assays.

##### Total Biomass Quantification

The total biofilm biomass was quantified using a crystal violet (CV) staining methodology [21]. For that, 24 h pre-formed biofilms were washed and firstly fixed with 500 µL of methanol, for 15 min. After the methanol removal, the biofilms were dried at room temperature, and then 500 µL of CV (1% *v/v*) added to each well. The stain was aspirate after 5 min and its excess was removed by washing the biofilms twice with sterile ultra-pure water. Finally, 500 µL of acetic acid (33% *v/v*) was added to each well to release and dissolve the CV stain. The absorbance of the CV solutions was then measured, at 570 nm and the results presented as absorbance per unit area (Abs CV cm^−2^). All experiments were performed in triplicate and in a minimum of three independent assays.

##### Filaments Enumeration

In order to quantify the number of cells in filamentous form state, pre-formed biofilms were scraped from the microtiter plate´s wells with PBS and the suspensions were sonicated for 10 s at 30% (Ultrasonic Processor; Cole-Parmer, Illinois, USA) to disaggregate the cells from matrix [11]. Then, aliquots of these suspensions were used to determine the percentage of cells as filamentous forms as describe above in Section 2.3.4. All experiments were performed in triplicate and in a minimum of three independent assays.

### 2.5. Statistical Analysis

Data were expressed as the mean ± standard deviation (SD) of a least three independent experiments. All the results were statistically analyzed using the GraphPad Prism 6 software. All tests were performed with a confidence level of 95%.

## 3. Results

### 3.1. Candida albicans Planktonic Growth on Artificial Urine and Saliva

**Figure 1:**
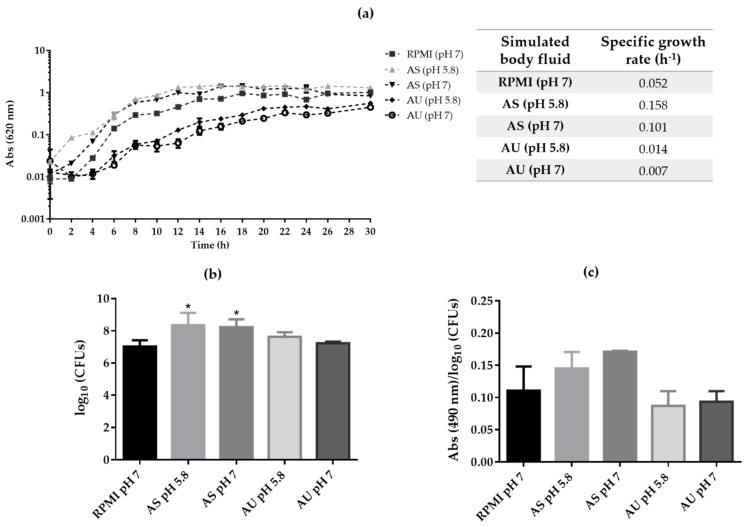
*Candida albicans* planktonic growth on simulated human body fluids. (**a**) Growth curves and specific growth rates over 30 h (**b**) Number of cultivate cells (log_10_ colony forming units (CFUs) and (**c**) Metabolic activity determination by XTT reduction (Abs 490 nm/log_10_ CFUs) obtained for planktonic growth of *C. albicans* SC5314 in Roswell Park Memorial Institute (RPMI) pH 7, artificial saliva (AS) (pH 5.8 and pH 7) and artificial urine (AU) (pH 5.8 and pH 7), at 24 h. Error bars represent standard deviation. * Significant difference between RPMI and simulated fluid (*p*-value < 0.05).

In order to analyze the ability of *C. albicans* free-cells to grow on simulated human body fluids, the optical density of free-floating cells cultivated in AS (pH 5.8 and 7) and AU (pH 5.8 and 7) was monitored for 30 h. Additionally, as a control, *C. albicans* cells were also grown in RPMI (pH 7). Figure 1a shows that *C. albicans* is able to grow on both simulated human body fluids tested, presenting the expected growth stages. However, the planktonic growth was slightly higher in AS, compared to AU and RPMI. For the same simulated fluid, it was possible to observe that the planktonic growth was slightly different depending on pH. Consistently, the specific growth rate values were higher at pH 5.8 in AS (0.158 h^−1^) and AU (0.014 h^−1^) and lower at pH 7 in AS (0.101 h^−1^) and AU (0.007 h^−1^) (Figure 1a).

Figure 1b presents the number of CFUs in the different simulated body fluids after 24 h. Planktonic growth presented higher number of CFUs in AS than in AU, even more than in the biofilms formed in RPMI (pH 7), which was statistically different (*p*-value < 0.05). The metabolic activity of planktonic cells was obtained through the XTT reduction assay and normalized by the number of CFUs obtained (Figure 1c). It was clear that planktonic metabolic activity was dependent on the fluid and on the pH. In fact, *C. albicans* cells’ planktonic growth presented a higher metabolic activity in AS than in AU despite not statistically different (*p*-value > 0.05). In the case of AS, the metabolic activity was higher than in RPMI, with higher values at pH 7 than in pH 5.8, however without statistical differences. Regarding AU, the metabolic activity was similar for the both pH tested, however it was slightly lower than RPMI (*p*-value > 0.05).

### 3.2. Candida albicans Biofilm Formation on Artificial Urine and Saliva

**Figure 2:**
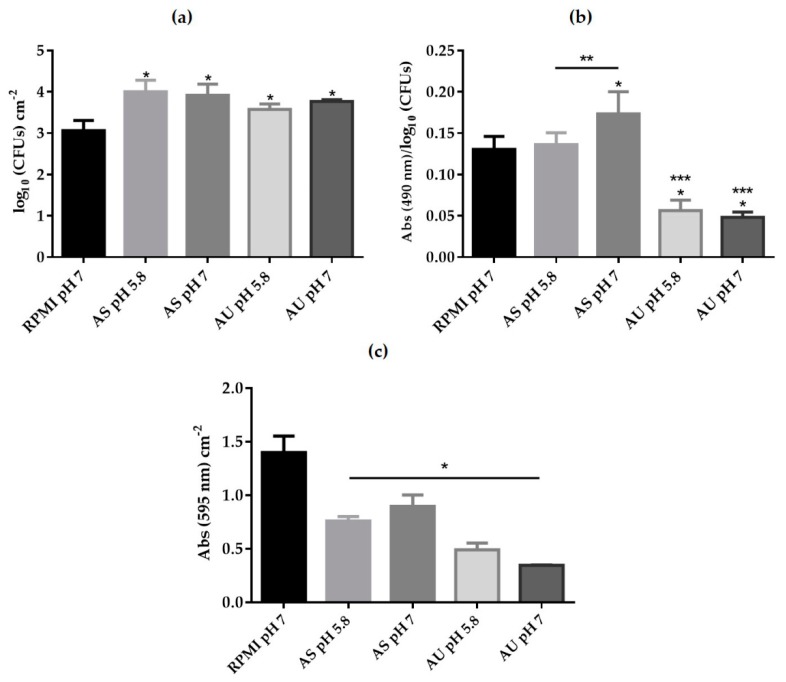
*Candida albicans* biofilm formation on simulated human body fluids. (**a**) Cultivate cells enumeration (log_10_ CFUs cm^−2^); (**b**) Metabolic activity determination by XTT reduction (Abs 490 nm/ log^10^ CFUs) and (**c**) Total biomass quantification (Absorbance CV cm^−2^) of *C. albicans* SC5314 of formation in RPMI (pH 7), AS (pH 5.8 and 7) and AU (pH 5.8 and 7) developed over 24 h. Error bars represent standard deviation. * Significant differences between RPMI and simulated fluid (*p*-value < 0.05); ** Significant differences between pH at the same body fluid (*p*-value < 0.05); *** Significant differences between AS and AU for the same pH (*p*-value < 0.05).

In order to determine the ability of *C. albicans* to develop biofilms on simulated body fluids, biofilms were formed in AS (pH 5.8 and 7) and AU (pH 5.8 and 7) over the 24 h. The biofilms were analyzed in terms of number of CFUs (Figure 2a), metabolic activity (Figure 2b) and total biomass (Figure 2c). As in planktonic studies, RPMI (pH 7) medium was used as a control. It was clear, that *C. albicans* was able to form biofilms in AS and AU at different pH, but the influence of simulated human body fluids on biofilm formation was evident. In fact, *C. albicans* biofilms presented statistically higher number of cultivable cells in both tested fluids (AS and AU) than in RPMI after 24 h of maturation (Figure 2a) (*p*-value < 0.05).

Figure 2b showed the metabolic activity values of pre-formed biofilms and it was also clear that metabolic activity was dependent on body fluid and pH. It was possible to observe a higher metabolic activity in AS than in AU, even more than in the biofilms formed in RPMI (pH 7) (*p*-value < 0.5). Furthermore, biofilms on AS at pH 7 presented metabolic activity values statistically higher than on AS at pH 5.8 and RPMI (pH 7) (*p*-value < 0.05), which was also observed in the planktonic cell growth (Figure 1c). In contrast, in the case of AU, for both pH, this activity was statistically lower when compared to RPMI (pH 7) (*p*-value < 0.05). Comparing AU at different pH, a slight decrease of metabolic activity from pH 7 to pH 5.8 (*p*-value > 0.05) can be seen (Figure 2b). Moreover, comparing both simulated body fluids at the same pH, it was possible to conclude that metabolic activity in AS (pH 5.8 and pH 7) was statistically higher than AU (pH 5.8 and pH 7), respectively (*p*-value < 0.05).

Consistently, *C. albicans* biofilms formed in AS presented higher biomass at 24 h than on biofilms formed in AU in both pH (Figure 2c). Moreover, comparing AS at different pH, the total biomass was slightly higher at pH 7 than pH 5.8, and in the case of AU, at pH 7 was slightly lower than pH 5.8, however without statistically significant differences between them. The value of total biomass obtained for the biofilms developed in RPMI (pH 7) was statistically superior to the value observed for both simulated human body fluids (*p*-value < 0.05) (Figure 2c).

### 3.3. Candida albicans Filamentation on Artificial Urine and Saliva

**Figure 3:**
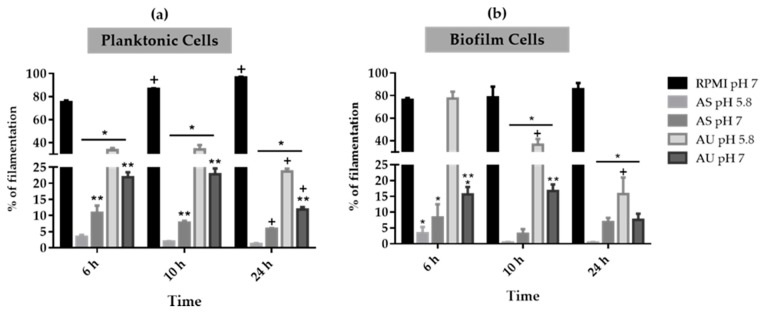
*Candida albicans* planktonic and biofilms cells filamentation on simulated human body fluids. Filamentous forms percentage of *C. albicans* SC5314 cells grown in (**a**) planktonic and (**b**) biofilm lifestyle in RPMI (pH 7), AS (pH 5.8 and 7) and AU (pH 5.8 and 7) over 24 h. * Significant differences between RPMI and simulated fluids at the same time point (*p*-value < 0.05); ** Significant differences between pH at the same body fluid (*p*-value < 0.05); **^+^** Significant differences for the same simulated fluid at different time points (*p*-value < 0.05).

*Candida albicans* cells grown in planktonic and biofilm lifestyles on RPMI (pH 7), AS (pH 5.8 and 7) and AU (pH 5.8 and 7) were analyzed by optical microscopy in order to evaluate their filamentous forms´ development (Figure 3). *Candida albicans* presented a statistically higher percentage of filamentous cells in RPMI (pH 7) in comparison to the percentages observed in AS and AU (*p*-value < 0.05), with exception of AU (pH 5.8) after 6 h of incubation in biofilm lifestyle. In RPMI (pH 7), *C. albicans* cells reached approximately 100% of filamentation over 24 h of grown in both lifestyles, which justifies the highest values on total biomass observed in Figure 2c. The results also revealed that *C. albicans* cells have a higher ability to develop filamentous forms on AU than in AS. However, in the case of AS, *C. albicans* presented a greater ability to develop filaments at pH 7 than at pH 5.8, with statistical differences in planktonic cells at 6 h and 10 h of incubation (*p*-value < 0.05) (Figure 3a). In contrast, the ability of *C. albicans* to filament in AU was higher at pH 5.8 compared to pH 7, with statistical differences in planktonic cells for all points and in biofilm cells at 6 h and 10 h of incubation (*p*-value < 0.05). Moreover, comparing the results in same fluid, it was possible to observe a decrease in percentage of *C. albicans* filaments over the time. In the case of planktonic cells, the percentage of filamentous forms in AS (pH 7) and AU (pH 5.8 and 7) was higher at 6 and 10 h of incubation than 24 h (*p*-value < 0.05) (Figure 3a). However, in biofilm lifestyle, the number of filaments was higher at 6 h of incubation than 10 h and 24 h, except in the case of AU (pH 5.8) (*p*-value < 0.05) (Figure 3b).

**Figure 4:**
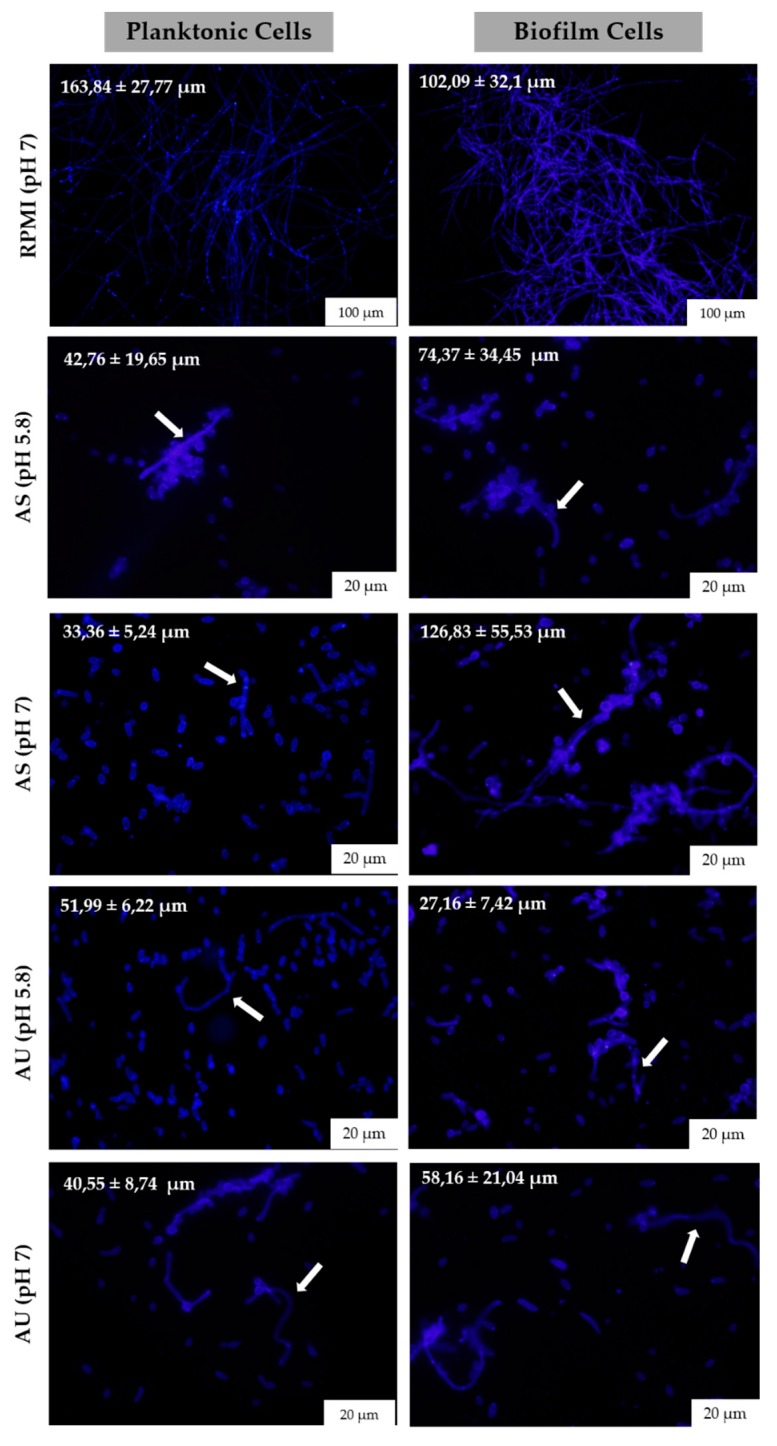
*Candida albicans* planktonic and biofilms cells filamentation on simulated human body fluids. Fluorescence microscopy images of *C. albicans* SC5314 grown in planktonic and biofilm lifestyle in RPMI (pH 7), AS (pH 5.8 and 7) and AU (pH 5.8 and 7) at 24 h. Original magnification was 10× for RPMI and 40× for simulated fluid. Arrows highlight filamentous forms.

The filamentous forms´ lengths presented in the different simulated body fluids were determined after fluorescence microscopy observation (Figure 4). It was clear that the filaments’ length was much higher in RPMI in both lifestyles compared to simulated body fluids. In other instances, on simulated body fluids the results showed it was verified differences between biofilm and planktonic growth lifestyles in most cases. In the case of AS at both pH, the filaments size on biofilm were much higher (AS pH 5.8 (74.37 ± 34.45 μm); AS pH 7 (126.83 ± 55.53 μm)) than in planktonic growth (AS pH 5.8 (42.76 ± 19.65 μm); AS pH 7 (33.36 ± 5.24 μm)), with more evidence at pH 7 (Figure 4). In contrast, in the case of AU pH 5.8, the opposite was observed, with the planktonic cells presenting a larger size (51.99 ± 6.22 μm) compared to biofilm cells (27.16 ± 7.42 μm). Concerning the results of AU pH 7, the filament size was similar between planktonic and biofilm cells (Figure 4).

## 4. Discussion

*Candida albicans* remains as the most prevalent of all *Candida* species with a range of incidence of around 40% [1,4]. Additionally, *C. albicans* pathogenicity has been attributed to several virulence factors, such as the ability to evade host defences; adherence; biofilm formation and the development of filamentous forms [11,22]. *Candida albicans* has been described as able to colonize and infect distinct sites including the urinary and oral tract [6,7,8]. Several environmental conditions, such as pH, allow *C. albicans* to take advantage of impaired immunity in immunocompromised patients and therefore facilitate the establishment of candidiasis [4,23]. In order to deepen the knowledge about the adaptability of *C. albicans* to different human body niches, the present work aimed to study the ability of *C. albicans* to grow as free cells, to form biofilm communities and to develop filamentous forms on two simulated human body fluids namely saliva and urine at distinct pH values.

The results revealed that, *C. albicans* is able to grow as free cells in the all fluids tested, however with different growth rates (Figure 1a). Planktonic growth was slightly higher in AS, compared to AU and RPMI. In the case of AS, the planktonic growth of *C. albicans* was slightly higher at pH 5.8 than pH 7 and even more than was observed in the control medium (RPMI pH 7). In contrast, the planktonic growth in urine was slower than in the saliva and RPMI and it was observed that at pH 5.8, the growth rate was slightly higher than pH 7. No correlation was observed between CFUs counts (Figure 1b) and the metabolic activity (Figure 1c) results with a slight increase in values of AS in comparison with AU and RPMI values.

A major virulence attribute of *C. albicans* is its ability to form biofilms, densely packed communities of cells adhered to a surface [9,11,22]. *Candida albicans* forms are highly structured and composed of multiple cell types (round budding yeast form cells and elongated hyphal cells) encased in an extracellular matrix [9,22,24,25]. In Figure 2, it was possible to observe that *C. albicans* was able to form biofilms on both simulated body fluids at both pH levels, however with different extensions. It was observed that *C. albicans* formed a stronger biofilm in AS than in AU, with highest metabolic activity (Figure 2b) and total biomass (Figure 2c). As in planktonic growth, no correlation was observed between CFUs number (Figure 2a) and biofilm cells metabolic activity (Figure 2b) at least in the case of AS. Despite *C. albicans* biofilms had a lower metabolic activity in AS at pH 5.8, these biofilms had a similar number of CFUs when compared to AS at pH 7. However, in the case of AU, the metabolic activity was similar for both pH, as well as the CFUs. The differences observed between both simulated body fluids can be explained by the presence of different components, such as a higher quantity of glucose in the case of saliva, which promotes the growth of *C. albicans* as free cells and as biofilm communities [26]. The higher values of glucose on AS may be the justification for the superior ability of *C. albicans* biofilm formation in this human body fluid (Figure 2). So far, a variety of indirect methods have been described for in vitro biofilms characterization and another important aspect raised by this study is that future research needs to consider which is the most appropriate parameter to investigate in vitro models since total biomass quantification, biofilm activity or cultivable cells do not necessary reflect the behaviour of the biofilm itself.

Besides biofilm formation, the ability to switch from yeast to filamentous forms is becoming one of the most alarming virulence factors associated to *C. albicans* infections [22]. In order to infer about the ability of *C. albicans* to filament on simulated body fluids, the numbers of filaments were enumerated in planktonic and biofilm growth lifestyles. Figure 3 reveals that the capability of *C. albicans* to filament was different in the two simulated body fluids. In the case of saliva, *C. albicans* presented a low rate of filamentation in both lifestyles (Figure 3), however, it showed an increased rate on the length of filaments in biofilm state (Figure 4). In urine, *C. albicans* cells presented higher numbers of filamentation than in saliva in biofilm lifestyle, despite presenting a decrease over the time. Moreover, in the case of saliva, *C. albicans* showed more ability to develop filamentous forms at pH 7 than pH 5.8, for both lifestyles. In contrast, in urine, *C. albicans* showed a higher ability to develop filaments at pH 5.8 than pH 7, in both lifestyles. In fact, it was described that the change of environmental pH is one of the major challenges often encountered by *C. albicans* making it able to colonize organs in humans with a wide range of pH conditions [27,28]. It has been well investigated that pH controls yeast in filamentous growth transitions [27,29,30], where acidic pH represses the yeast in filamentous growth transition, while neutral and alkaline conditions promote filamentation [27,29]. These results highlight *C. albicans* genome plasticity that rapidly generates diversity in response to different environmental human body niches resulting in biofilm formation and/or filaments development, as has been described in other adaptive processes in fungi [12,13,31,32].

## 5. Conclusions

Infections caused by *C. albicans* are associated with a high mortality and morbidity rate, and their ability to adapt to the different niches of the human body has been shown to be essential for the survival and virulence of these commensals´ pathogens. From this study, it was possible to conclude that *C. albicans* presented a high plasticity and adaptability to different human body fluid at different pH levels, resulting in high ability to grow as free cells or as biofilm communities composed with yeast and elongated hyphal cells. This may be the justification for the high number of the oral and urinary candidiasis in the whole world.
